# Identification of reference genes for real-time PCR cytokine gene expression studies in sheep experimentally infected with *Fasciola hepatica*

**DOI:** 10.1038/s41598-018-37672-7

**Published:** 2019-02-06

**Authors:** I. L. Pacheco, N. Abril, R. Zafra, N. Morales-Prieto, V. Molina Hernández, M. T. Ruiz, R. Perez-Caballero, A. Martínez-Moreno, J. Pérez

**Affiliations:** 1 Department of Anatomy and Comparative Pathology, University of Córdoba, Córdoba, Spain; 2 Department of Biochemistry and Molecular Biology, University of Córdoba, Córdoba, Spain; 3 Department of Animal Health, Parasitology section, University of Córdoba, Córdoba, Spain

## Abstract

The aim of this study was to validate reference genes for gene normalisation using qRT-PCR in hepatic lymph nodes (HLN) and livers from sheep infected with *Fasciola hepatica* during early and late stages of infection. To this end, a comprehensive statistical approach (RefFinder) encompassing four different methods of analysis (geNorm, BestKeeper, ΔCt method and NormFinder) was used to validate ten candidate reference genes. Stability analysis of gene expression followed by pairwise variation (Vn/Vn + 1) analysis revealed that PGK1, HSP90AA1 and GYPC were the most stable reference genes and suitable for qRT-PCR normalisation in both HLN and liver tissues. These three genes were validated against FoxP3, IL-10, TGF-β, TNF-α and IL-1β genes in the HLN tissue of sheep vaccinated with Cathepsin L1 from *F. hepatica* and unvaccinated infected and uninfected controls during early stages of infection. In the liver, the three reference genes were validated against TNF-α and IL-1β during chronic stages of infection with *F. hepatica* and in uninfected controls. Our study is the first to evaluate and validate sheep reference genes in order to provide tools for monitoring cytokines in *Fasciola hepatica* infected sheep target organs. Our results present an approach to elucidate the role of different cytokines in *F. hepatica* vaccinated and infected sheep.

## Introduction

*Fasciola hepatica* is the causative agent of fasciolosis in temperate climates, infecting a wide range of hosts — particularly ruminants — and resulting in estimated economic losses of US $3,200 million per annum worldwide to the agricultural sector^1^. It is also a major human pathogen and fasciolosis is considered an emerging zoonosis in areas of Africa, Asia and Latin America where the disease is endemic in domestic species^2^. Current control of fasciolosis in ruminants is based on the use of anthelmintic drugs, but such methods are expensive and may lead to the development of drug resistance^3,4^ and increased risks of chemical residues in milk and meat. Vaccines are an environmentally friendly alternative for the control of fasciolosis, hence, there is increasing interest in the development of vaccines which protect against this disease in ruminants. Although several vaccine candidates have shown promising results in ruminant or laboratory animals, to date none have shown sufficiently consistent protection to be taken to pre-commercial development^5–7^. The relatively slow progress in vaccine development is due at least in part to the high immunomodulatory properties of *F. hepatica*^8,9^.

Understanding the mechanism of the host immune response is crucial in order to enhance vaccine efficacy. To this end, some studies have evaluated the Th1 and Th2 responses and the mechanism by which *F. hepatica* polarises the immune response to a non-protective Th2 response in the early stages of infection^10^. Other studies have focused on the expression of regulatory cytokines such as TGF-β and IL-10^11^ and transcription factors such as FoxP3^8^. These proteins have been identified as key in the regulation of the host immune response and therefore influence the Th1 and Th2 responses.

Since cytokine proteins are labile gene expression is an appropriate method to measure cytokine levels in different conditions. The process of gene expression translates gene-encoded information into final functional gene products, such as a proteins or non-coding RNA. Quantitation of the expression of key genes is fundamental for understanding the molecular, genetic and functional mechanisms of disease. Quantitative real-time polymerase chain reaction (qRT-PCR) is efficient, sensitive and probably the most reliable approach for accurate quantification of gene expression from different sources and samples. This methodology, being relatively simple and cost-effective, is considered the gold standard for measuring the copy number of specific targets^12^.

Quantitative RT-PCR is greatly affected by RNA purity and integrity, genomic DNA contamination, pipetting errors, concentration and primer efficiencies. The generation of precise, reproducible and accurate results requires qRT-PCR experiments to be carried out following the Minimum Information for the Publication of Quantitative Real-time PCR Experiments (MIQE) guidelines^13–15^. The choice of reference genes used for standardisation is critical, and they must be validated within the context of each individual experimental setup if data are to be biologically meaningful^15^.

Recently, some studies have been published focusing on the identification and selection of sheep reference genes in abomasum, skin and leukocyte cells^16–18^. To the best of our knowledge, no study has addressed the selection of suitable reference genes for *F. hepatica* in liver and lymph nodes tissues. The present study sought to determine the expression stability of 10 commonly used reference genes in order to identify those most suitable for qRT-PCR normalisation in experiments performed on liver and lymph node tissues of *F. hepatica*-infected sheep. The expression profiles of putative reference genes (PGK1, B2M, RPLP0, G6PD, SDHA, ACTB, HSP90AA1, GYPC, GUSB and TUBB) were evaluated using RefFinder software^19^ that integrates BestKeeper^20^, Normfinder^21^, geNorm^22^ and comparative ∆Ct methods^23^ to produce a comprehensive ranking of genes confirmed by the use of multiple algorithms. Changes in expression patterns of FoxP3, IL-10, TGF-β, TNF-α and IL-1β in the hepatic lymph nodes (HLN) of vaccinated sheep infected with *F. hepatica*, and TNF-α and IL-1β in the liver of sheep unvaccinated and infected with *F. hepatica* were investigated using the selected reference genes, PGK1, HSP90AA1 and GYPC.

The aim of this work was to find valuable reference genes for future gene expression studies in both HLN and liver samples. Since it would provide increased insight into the molecular mechanisms of sheep immune responses, revealing approaches with which to boost the immune response using vaccines and prevent *F. hepatica* infection in sheep.

## Results and Discussion

In order to identify a set of reference genes suitable for use under different experimental conditions in two key immunological tissues (HLN and liver), we performed stability studies on a select group of putative reference genes. With the aim of finding vaccines to control infections caused by *F. hepatica* in sheep, two separate experiments were performed according to MIQE Guidelines^13^.

### Quality and integrity of RNA samples

**Figure 1:**
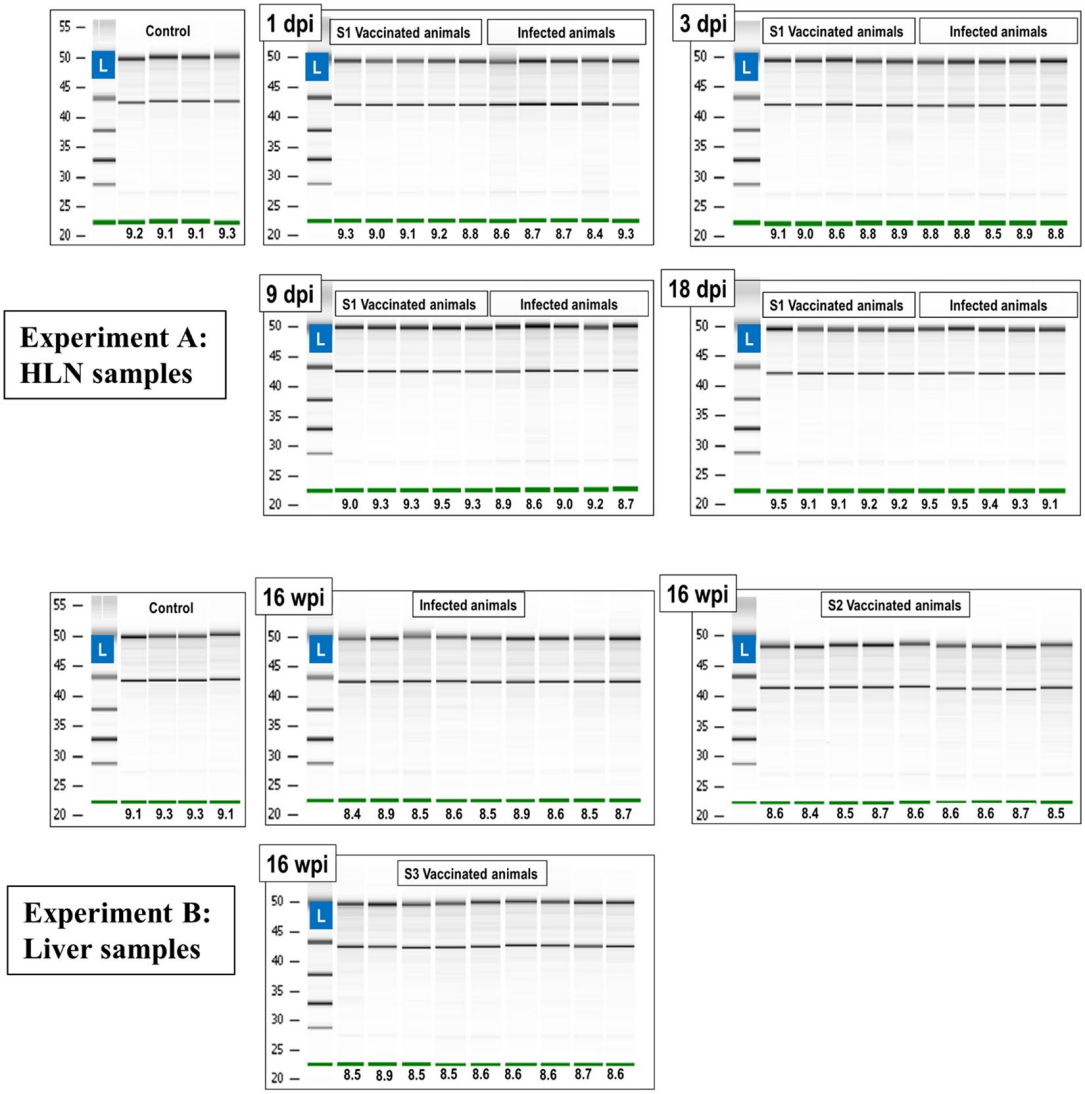
RNA integrity. Gel images of RNA samples and the RIN values (under the green lines) obtained by the Agilent Bioanalyzer. RIN = RNA Integrity Number; L = RNA Ladder; dpi: days post-infection; wpi: weeks post-infection. Green line in: Bioanalyzer internal marker.

To ensure sample quality and integrity, RNA was extracted from frozen tissue and treated with DNase-I to avoid amplification of residual genomic DNA (gDNA). The A260/A280 and A260/A230 absorbance ratios were measured and RNA preparations deemed to be acceptably pure when ratios were close to two. The integrity of RNA is also critical for reliable measurement of gene expression data^24^. Evaluation of RNA integrity carried out by determination of the ratio of 28S:18S ribosomal RNA using agarose gel electrophoresis has been shown to be unreliable. The Agilent 2100 bioanalyser is an automated bio-analytical device which uses microfluidic technology for electrophoretic separation of RNA molecules in an automated and reproducible manner^25^. Results are visualised as electropherograms which are used to calculate an RNA integrity number (RIN) for each sample. Numerical values of RINs range from 10 (intact) to 1 (totally degraded). As shown in Fig. 1, the RIN values of all samples in this study exceeded 8.0, indicating that our total RNA samples were of sufficient quality.

### Amplification specificity and primer efficiency

**Figure 2:**
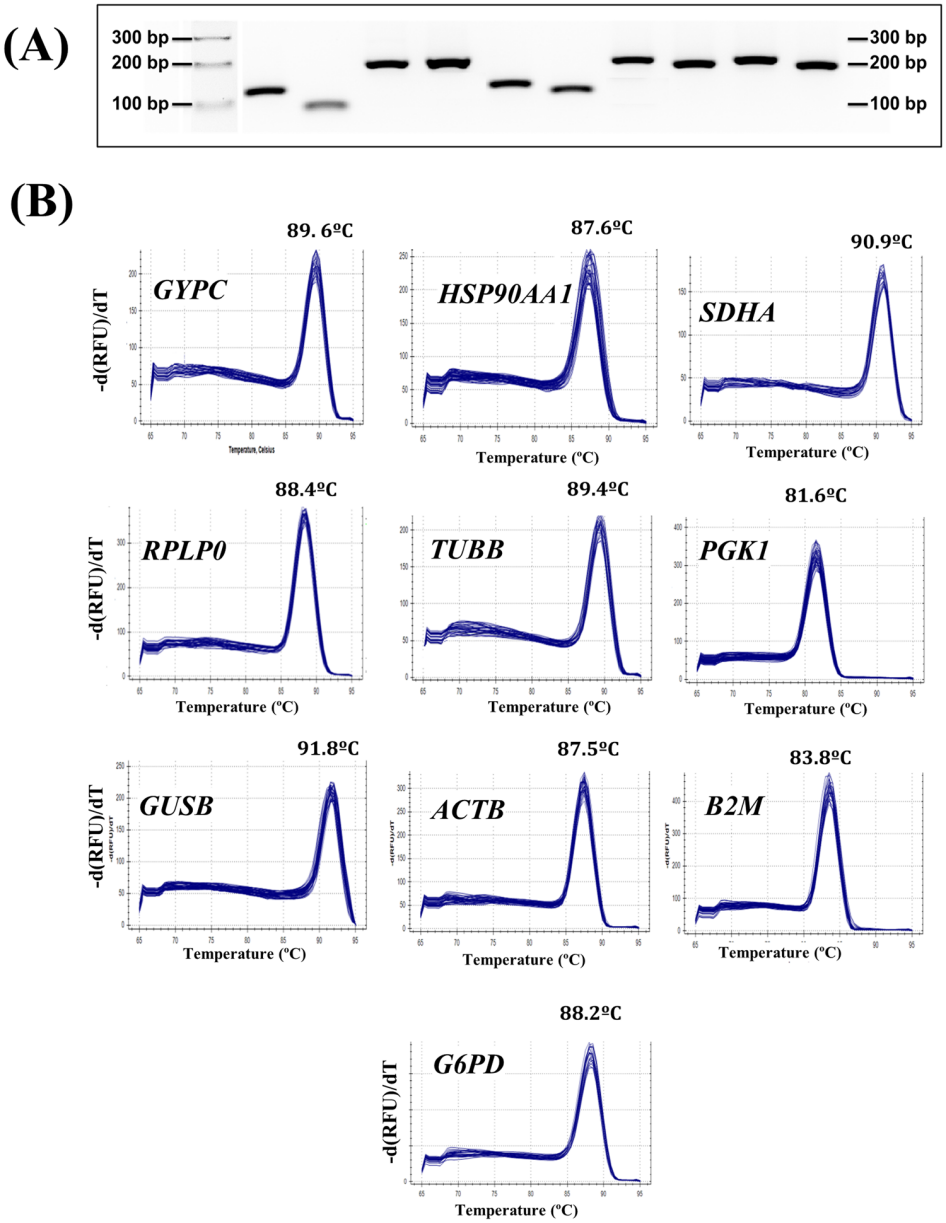
Confirmation of gene specificity and amplicon size. (**A**) Agarose gel of qRT-PCR products for each gene with the expected size. Equal amounts of cDNA from any animal were mixed and amplified with each primer pair and PCR products loaded on a 2% agarose gel. A standard DNA molecular weight ladder was loaded. Gel has been cropped to show only relevant information; the full-length gel is shown in Supp. Figure 1. (**B**) Melting curves of the 10 putative reference genes showing a single peak. The Tm values of each amplification product are shown over the peaks.

Primer selection is critical to ensure specific and efficient amplification of selected targets. We used previously reported primer pairs or newly designed primer sets to amplify an amplicon of 100–200 bp (Table 1, Fig. 2) of ten putative reference genes. Primers free of hairpins and duplexes, and with a high melting temperature (Tm; >68 °C, Table 1) were used. A two-step PCR protocol was used, where primer/target hybridisation and polymerase extension occur at 68/70 °C, ensuring specificity of amplification. A single band of the expected size on agarose gel and a single peak on the qRT-PCR melting curves (Fig. 2) confirmed that each primer pair amplified a unique product, no primer-dimers were generated and none of the transcript contained tissular or experimentally-induced alternative splicing in the primer hybridisation zone. The Tm of all the PCR products ranged from 81.6 °C (PGK1) to 91.8 °C (GUSB). Products were sequenced and showed 100% identity with the fragment sequences on which primer design was based.

**Table 1:** Primers used in this work.

Target^a^	Accesion number^a^	Primers sequences^b^ 5′–3′	Primers Tm (°C)^c^	Amplified product length (bp)^d^	Amplification efficiency	References
					(R^2^)^e^	
*TNF-α*	NM_001024860.1	F 5′-CCACGCTCTTCTGCCTGCTGCACTTCGG-3′	73.1	146	0.996	Pacheco *et al*.^33^
		R 5′-AACGTGGGCTACCGGCTTGTTATTTGAGGC-3′	73.6		(99.56)	
*IL-1β*	NM_001009465.2	F 5′-GAAGCTGAGGAGCCGTGCCTACGAACA-3′	68.8	185	1.001	Pacheco *et al*.^33^
		R 5′-CCAGCACCAGGGATTTTTGCTCTCTGTCC-3′	69.0		(99.85)	
*FoxP3*	NM_001144947.1	F 5′-GCCCATCTGGCTGGGAAGATGGCCCAAACC-3′	76.5	166	0.991	Pacheco *et al*.^33^
		R 5′- AGAGGTGCCTCCGCACGGCAAACAGG-3′	76.2		(99.16)	
*IL-10*	NM_001009327.1	F 5′-TCAGCCGTGCTCTGTTGCCTGGTCTTCC-3′	73.8	124	0.999	Pacheco *et al*.^33^
		R 5′- GGACGTCCCGCAGCATGTGGGGCAG-3′	73.5		(99.9)	
*TGF-β*	NM_001009400.1	F 5′- GGGCTTTCGCCTCAGTGCCCACTGTTC-3′	73.8	151	1.009	Pacheco *et al*.^33^
		R 5′- CAGAGGGGTGGCCATGAGGAGCAGG-3′	73.8		(99.9)	
*PGK1*	NM_001142516.1	F 5′-GTGAAGGGGAAGCGGGTCGTCATGAGAG-3′	72.9	99	1.002	Pacheco *et al*.^10^
		R 5′-GCTTGGAACAGCAGCCTTGATCCTCTGG-3′	72.1		(99.43)	
*B2M*	NM_001009284.2	F 5′-CAAGACACCCGCCAGAAGATGGAAAGC-3′	71.6	180	0.989	Pacheco *et al*.^10^
		R 5′-GGAGTGAACTCAGCGTGGGACAGAAGG-3′	70.3		(99.91)	
*RPLP0*	XM_004017413.2	F 5′-CGGCTGCTGCCCGTGCTGGTGCCAT-3′	77.7	191	1.002	Pacheco *et al*.^10^
		R 5′-TTCGCTGGCGCCCACCTTGTCTCCGGTC-3′	77.1		(99.81)	
*G6PD*	NM_001093780.1	F 5′-CGGGCGAGAGCAACGAAGCACAGAGAGC-3′	74.0	161	1.004	Pacheco *et al*.^10^
		R 5′-CCAGGTCCCCCGATGCACCCATGATG-3′	74.2		(99.82)	
*SDHA*	XM_012097183.1	F 5′-CCATGAGTTTGATGCCGTGGTGGTCGGTGC-3′	70.7	184	1.006	Pacheco *et al*.^10^
		R 5′-CCGCCAGTTGTCCTCCTCCATGTTCCCCA −3′	70.4		(99.51)	
*ACTB*	NM_001009784.1	F 5′-GCGTGATGGTGGGCATGGGCCAGAAGG-3′	76.0	170	1.003	This work
		R 5′-GGGGGCCACACGCAGCTCGTTGTAGAAGG-3′	75.7		(99.99)	
*HSP90AA1*	XM_004017995.3	F 5′-GCCGCCCCTGGAAGGAGACGACGACACG-3′	71.1	130	1.005	This work
		R 5′-GCCAGGCGAGCCTCGGCAGCGCTCA-3′	72.0		(99.53)	
*GYPC*	XM_004004772.3	F 5′-TGCCCCGCACCGGCCAGCGATGAG-3′	79.2	110	1.006	This work
		R 5′-GGTCGGGCTCTGGGGTCCAAAGGGGCGTC-3′	79.3		(99.85)	
*GUSB*	XM_015103904.1	F 5′-CTTCCGCGCCGACTTCTCCGACAAC-3′	72.4	185	1.004	This work
		R 5′-GCAGCGTGATCTCCCGTTCATACCACACC-3′	72.8		(99.89)	
*TUBB*	XM_012101347.2	F 5′-CCAGGACGCCACGGCCGAGGAGGAGGG-3′	79.1	112	0.997	This work
		R 5′-TGGCCGCAGCCGCGTCCCACCCCTTC-3′	79.8		(99.58)	

^a^Gene symbols and accession numbers are according to the NCBI Gene database, ^b^Sequence of forward (F) and reverse (R) primers, ^c^Melting temperature (°C) calculated by the Oligo 7 software, ^d^PCR product size in base pair (bp), ^e^The real-time PCR efficiencies (E) were calculated from each efficiency curve according to the equation E = 10^(−1/slope)^ −1. E is in the range from 0 (minimum value) to 1 (maximum and optimum). i.e. E = 1 is equal to 100% efficiency.

Replicate PCR reactions generated highly reproducible results with standard errors of the mean (SEM) values that represented less that 1% of the respective mean value. The efficiency of PCR is highly dependent on the primers used and presence of inhibitors in the sample which may be derived from regents used in the retrotranscription step. Table 1 provides — for each primer pair — the efficiency of PCR amplification (E) and coefficient of correlation (R^2^) derived from the slope of the standard curves spanning seven orders of magnitude from 2 to 2 × 10^5^ pg of cDNA per reaction. Amplification efficiency varied from 98.9% for B2M to 100.6% for SDHA, and R^2^ values were >99.4% in all cases (Table 1).

### Comparison of expression stabilities of candidate reference genes by descriptive statistics

Reference genes are used in qRT-PCR experiments to normalise data by correcting for differences in quantities of template cDNA. A perfect reference gene shows no change in expression between the samples whether they come from different tissues, experimental conditions or time points. Reference genes must be carefully selected based on experimental data. Following the protocol in the MIQE guidelines^13,15,26^, equal amounts of cDNA from three or four animals per experimental group were pooled and used to determine Ct values for each candidate gene in both HLN and liver tissues. The Ct values (84 data per gene) were measured in triplicate and exported to an excel file provided in Supplementary Table S1. Expressions levels of the putative reference genes were analysed for the most abundant (B2M, mean Ct = 14.041 ± 0.79) to the least abundant and more variable gene (TUBB, mean Ct = 27.205 ± 3.13). Although the differences in Ct values between genes were minimal, PGK1 and SDHA showed more stable expression than other genes (Supplementary Table S1).

**Figure 3:**
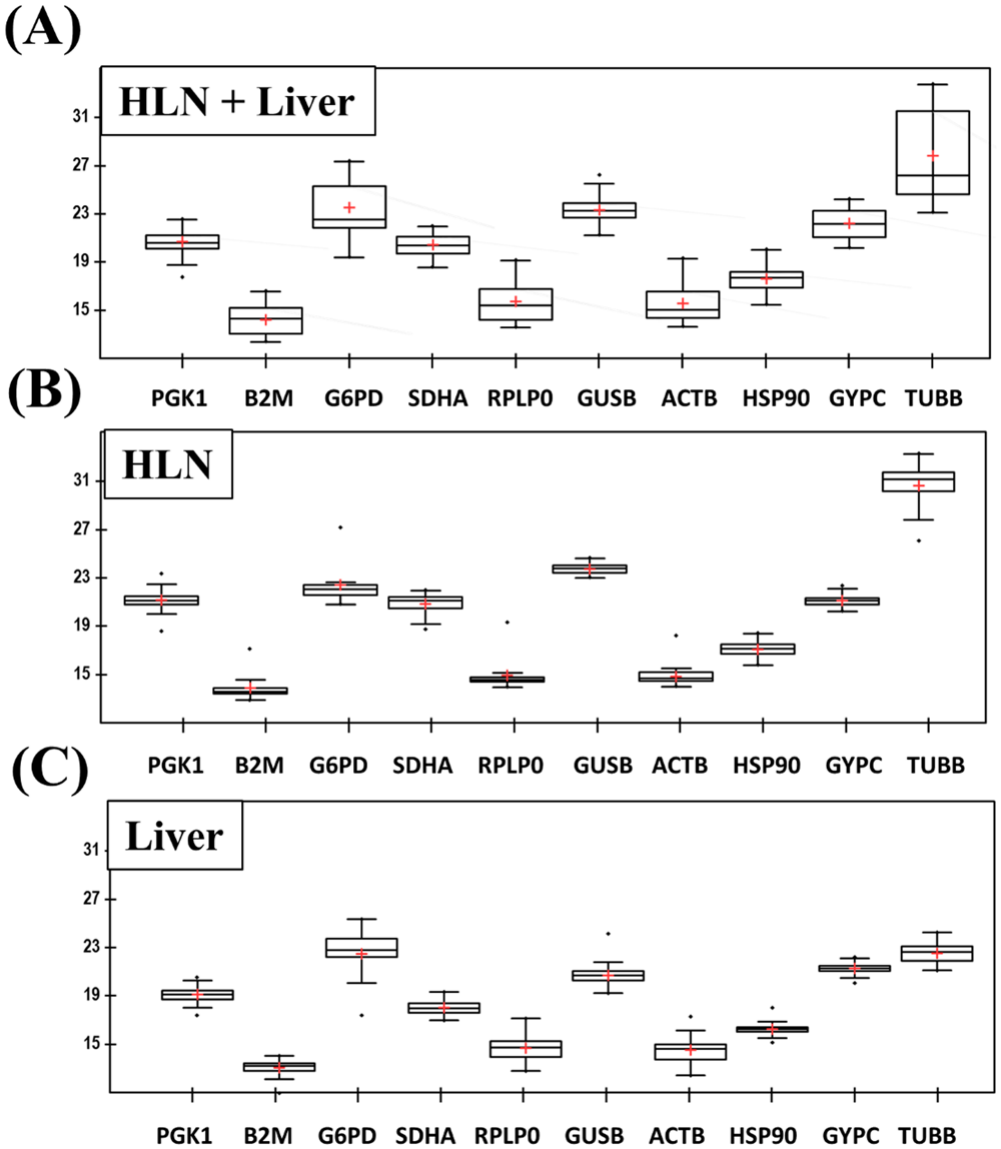
Distribution of Ct values for candidate reference genes. (**A**) Both tissues, HLN and liver Ct values were analyzed together. (**B**) HLN data analysis. (**C**) Liver data analysis. Boxes: range of Ct values; black center line: median Ct; cross: mean score; upper and lower hinges: 75 and 25 percentiles; whiskers: largest/smallest Ct values within a distance of 1.5 times IQR (Interquartile range) from the upper and lower hinges.

Descriptive statistics were performed with Excel complement XLStat *v*. 19.4.45191 software (Addisoft). The results are listed in Supplementary Table S2 and presented in Fig. 3.

Statistical parameters of the Ct values were calculated for HLN and liver samples together, HLN samples alone and liver samples alone. In all combinations, TUBB and G6PD had the most variable expression levels, indicated by high standard deviation (SD). All other genes showed remarkable stability in both HLN and liver tissues when analysed individually, with PGK1, HSP90AA1, GUSB and SDHA showing the highest stable expression levels when both tissues were analysed together. These four genes showed intermediate average Ct values in both tissues, making them suitable reference genes.

To definitively identify the most stable reference genes in both tissues, more complex analyses were performed using geNorm^22^, the comparative ∆Ct method^23^, NormFinder^21^, BestKeeper^20^ and RefFinder^19^. The web-based tool RefFinder integrates data generated by these four algorithms individually and produces an integrative overall ranking of stability. Genes with a lower ranking are associated with higher expression stability. Table 2 shows that most of the algorithms indicated that PGK1, HSP90AA1 and GYPC were the most stable genes under all experimental conditions and in both tissues.

**Table 2:** Stability ranking of candidate reference genes.

GeNorm		NormFinder		Bestkeeper		ΔCt Method		RefFinder	
*Gene name*	*Stability value*	*Gene name*	*Stability value*	*Gene name*	*Standard deviation*	*Gene name*	*Average SD*	*Gene name*	*Geomean of ranking values*
**HLN** + **Liver**									
**HSP90AA1**	0.608	**PGK1**	0.135	**PGK1**	0.630	**PGK1**	1.330	**PGK1**	**1.320**
**GYPC**	0.608	**HSP90AA1**	0.546	**B2M**	1.020	**HSP90AA1**	1.400	**HSP90AA1**	**2.000**
**PGK1**	0.741	**SDHA**	0.700	**G6PD**	1.760	**B2M**	1.440	**GYPC**	**3.460**
**B2M**	0.775	**B2M**	0.771	**SDHA**	0.710	**GYPC**	1.490	**B2M**	**3.940**
**ACTB**	0.812	**GUSB**	0.892	**RPLP0**	1.310	**ACTB**	1.530	**SDHA**	**4.740**
**RPLP0**	0.902	**GYPC**	0.935	**GUSB**	0.640	**RPLP0**	1.630	**GUSB**	**5.180**
**G6PD**	1.038	**ACTB**	0.941	**ACTB**	1.070	**SDHA**	1.640	**ACTB**	**5.920**
**SDHA**	1.168	**RPLP0**	1.190	**HSP90AA1**	0.710	**GUSB**	1.740	**RPLP0**	**6.930**
**GUSB**	1.261	**G6PD**	1.609	**GYPC**	1.060	**G6PD**	1.940	**G6PD**	**8.450**
**TUBB**	1.816	**TUBB**	3.970	**TUBB**	3.220	**TUBB**	4.040	**TUBB**	**10.000**
**HLN**									
**HSP90AA1**	0.420	**HSP90AA1**	0.393	**PGK1**	0.450	**HSP90AA1**	0.760	**HSP90AA1**	**1.000**
**GYPC**	0.420	**PGK1**	0.423	**B2M**	0.440	**PGK1**	0.780	**GYPC**	**2.060**
**PGK1**	0.530	**GYPC**	0.440	**G6PD**	1.340	**GYPC**	0.790	**PGK1**	**2.630**
**SDHA**	0.550	**B2M**	0.481	**SDHA**	0.450	**SDHA**	0.820	**B2M**	**4.160**
**B2M**	0.586	**SDHA**	0.510	**RPLP0**	0.780	**B2M**	0.830	**SDHA**	**4.470**
**GUSB**	0.644	**RPLP0**	0.530	**GUSB**	0.500	**RPLP0**	0.870	**GUSB**	**6.930**
**TUBB**	0.692	**ACTB**	0.618	**ACTB**	0.810	**ACTB**	0.920	**RPLP0**	**6.930**
**RPLP0**	0.738	**GUSB**	0.731	**HSP90AA1**	0.270	**GUSB**	0.950	**TUBB**	**7.940**
**ACTB**	0.774	**TUBB**	0.779	**GYPC**	0.330	**TUBB**	0.990	**ACTB**	**7.940**
**G6PD**	0.923	**G6PD**	1.434	**TUBB**	0.640	**G6PD**	1.520	**G6PD**	**10.000**
**Liver**									
**GUSB**	0.426	**GYPC**	0.294	**PGK1**	0.530	**GYPC**	0.920	**GYPC**	**1.000**
**GYPC**	0.426	**PGK1**	0.318	**B2M**	0.560	**GUSB**	0.940	**GUSB**	**1.860**
**ACTB**	0.533	**GUSB**	0.331	**G6PD**	0.950	**PGK1**	0.950	**PGK1**	**3.500**
**HSP90AA1**	0.564	**HSP90AA1**	0.516	**SDHA**	0.690	**HSP90AA1**	1.010	**ACTB**	**3.870**
**PGK1**	0.642	**ACTB**	0.554	**RPLP0**	0.780	**ACTB**	1.030	**HSP90AA1**	**4.000**
**SDHA**	0.684	**SDHA**	0.588	**GUSB**	0.330	**SDHA**	1.070	**SDHA**	**6.240**
**B2M**	0.758	**B2M**	0.666	**ACTB**	0.410	**B2M**	1.080	**B2M**	**6.740**
**RPLP0**	0.886	**RPLP0**	1.190	**HSP90AA1**	0.510	**RPLP0**	1.370	**RPLP0**	**8.000**
**G6PD**	0.984	**G6PD**	1.371	**GYPC**	0.330	**G6PD**	1.500	**G6PD**	**9.000**
**TUBB**	1.187	**TUBB**	1.926	**TUBB**	1.270	**TUBB**	2.000	**TUBB**	**10.000**

Expression levels of candidate reference genes were evaluated through determination of the Ct values. Data were analysed with the RefFinder tool, resulting in a comprehensive ranking of the studied genes (Table 2 in bold). Grey shadow indicates the rankings of PGK1, HSP90AA1 and GYPC — the three most stable genes according to RefFinder algorithm and therefore the selected reference genes for HLN and liver qRT-PCR studies.

### Determination of the optimal number of reference genes for normalisation

**Figure 4:**
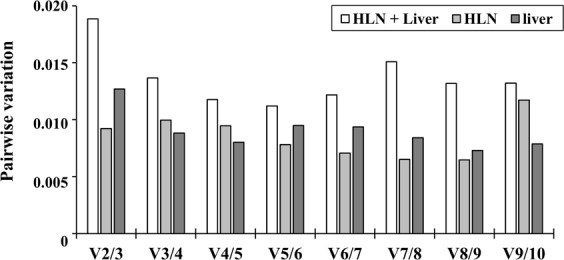
Determination of the optimal number of reference genes for normalization. The geNorm algorithm was used to determine the pair-wise variation value (Vn/n + 1) from the Ct values obtained in HLN, liver and both tissues altogether.

As a general rule, the number of reference genes used in an experiment is determined by the fold change of RNA of the samples that are being compared^15^. It is recommended to use at least two reference genes, since use of only one may lead to large errors. The pairwise variation (Vn/Vn + 1) is an index used to determine the minimum number of reference genes required for accurate qRT-PCR normalisation in gene expression studies. The geNorm algorithm was used to calculate the pairwise variation between sequential normalisation factors, with the recommended value of 0.15 being used as the cut-off for selecting a suitable number of reference genes. The V2/3 values of HLN and liver analysed individually or together were less than 0.15 (Fig. 4), which suggested that use of two reference genes would be sufficient for this study. However, because all of the different algorithms revealed that PGK1, HSP90AA1 and GYPC were the most stable genes, we selected all three as reference genes for normalisation of our qRT-PCR data to increase the resolution and accuracy of results^27^.

Other studies have assessed the stability of various genes frequently used in qRT-PCR in sheep^16,17,28,29^. Only one of these studies identified one of our selected reference genes (PGK1) as suitable for accurate and reproducible qRT-PCR analysis of gene expression in sheep. In the cited studies, authors used different tissues and a reduced number (no more than eight) of candidate genes with no more than two statistical approaches (usually geNorm and NormFinder). No statistical approach can cover all variables associated with gene expression studies; therefore, drawing conclusions based on one or two methods can lead to false positives and incorrect conclusions^30^. In our study, we used a comprehensive statistical approach (RefFinder) to determine good reference candidates for reliable normalisation of gene expression data in HLN and liver tissues of sheep. The benefits of using this statistical tool are evidenced by the consistency of the gene ranking with our previous results using only five putative reference genes^10^.

### Pathology

In experiment B, the uninfected control group (group five) showed no gross or histopathological hepatic changes. Gross changes in infected animals (group four) consisted of enlarged and whitish bile ducts and gall-bladder, and irregular 0.2–1.5 cm length scars mainly on the left hepatic lobe.

Histopathological changes in group four consisted of fibrous perihepatitis, chronic tracts composed of fibrosis, macrophages with hemosiderin pigmentation, severe periportal fibrosis and hyperplastic cholangitis with abundant infiltrate composed of lymphocytes, plasma cells and eosinophils. Granulomas with a necrotic centre surrounded by macrophages and lymphocytes were also found in the hepatic parenchyma and portal areas. Adult flukes and eggs were found within bile ducts.

### Reference gene validation

Five immune response related genes — TGF-β, IL-10, FoxP3, IL-1β and TNF-α — were selected as target genes to validate the reliability of identified reference genes. This study was divided in two different experiments which results are shown below.

**Figure 5:**
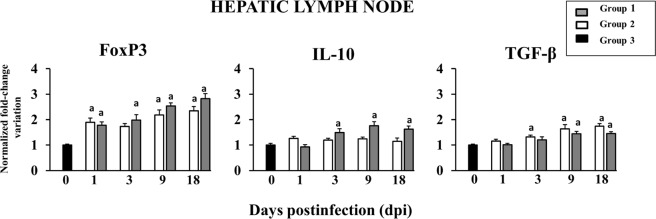
Gene expression of FoxP3 and regulatory cytokines in HLN. ^a^Indicates significant differences in comparison with group 3 (negative control).

Experiment A focused on HLN tissue as this is responsible for liver drainage, and it would be informative to elucidate changes that occur on a systemic level. The expression levels of FoxP3, IL-10 and TGF-β in HLN are illustrated in Fig. 5.

The expression of these three genes in group three increased gradually throughout the course of the experience. The expression of FoxP3 at 1 day post infection (dpi) was significantly higher in group one (p < 0.05) and group two (p < 0.01) than group three; and at 3 dpi it was only significantly higher for group one (p < 0.01). At 9 and 18 dpi the expression of FoxP3 in group one and two was about 1.5-fold and 2-fold higher than in group three respectively (p < 0.001). Throughout the experiment, the expression of FoxP3 in group one was higher than group two except at 1 dpi. This increase of FoxP3 gene expression agrees with the significant increase in numbers of abomasal FoxP3^+^ regulatory T cells (Tregs) seen in sheep infected with *Teladorsagia circumincta*^31^. Increase of FoxP3 expression has also been seen in experiments with *F. hepatica* and other helminths. A study carried out on mice immunised with *Schistosoma mansoni* egg antigens and subsequently subjected to induced colitis showed increased production of FoxP3^+^ Tregs and IL-10 compared with mice with induced colitis only. A positive correlation between FoxP3^+^ Tregs and IL-10 and consequent decrease in colitis was found^32^.

Expression of IL-10 increased significantly at 3 dpi (p < 0.05), 9 dpi (p < 0.001) and 18 dpi (p < 0.001) in group one compared with group three, although no significant differences were found between IL-10 expression in group two and group three at any point (Fig. 5). An increase in IL-10 expression has been seen to be associated with the expansion of FoxP3^+^ Tregs^31,33^. The expression of TGF-β showed a gradual increase throughout the time course of the experiment. At 3 dpi, group two showed significantly higher TGF-β expression than group three (p < 0.05); at 9 and 18 dpi, significant increases were seen in groups one (p < 0.001) and two (p < 0.001) compared with group three. Both IL-10 and TGF-β are important for regulation of immune responses and stimulation of the FoxP3 transcription factor^34^. A study in mice infected with the intestinal helminth *Heligmosomoides polygyrus* showed that a TGF-β mimic was present in excretory-secretory (ES) antigens, and an expansion of FoxP3^+^ Tregs was induced by ES antigens. This demonstrates that FoxP3 induction by ES antigens is dependent on TGF-β signalling^35^. The expression of IL-10 and TGF-β showed similar patterns throughout the course of infection, which correlates with the results of a study of HLN tissue of sheep in the chronic stages of infection — sheep with a heavy fluke burden showed significantly lower expression levels of these cytokines compared with sheep with a light fluke burden^36^. In our study, expression of these cytokines increased as infection progressed, which is in agreement with the upregulation reported in HLN^36^. Increased expression of regulatory cytokines supports a role for Tregs in the generation of a suppressive environment which is favourable to parasite persistence. Furthermore, TGF-β is an essential cytokine in the development of fibrosis, as demonstrated in our previous study^33^.

**Figure 6:**
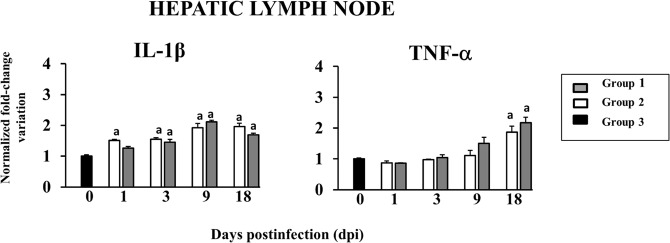
Gene expression of proinflammatory cytokines in HLN. ^a^Indicates significant differences in comparison with group 3 (negative control).

The expression of proinflammatory cytokines was also evaluated in experiment A. The results of TNF-α and IL-1β analyses are summarised in Fig. 6. A gradual increase in IL-1β levels was observed from 1 to 9 dpi in groups one and two compared with group three, with slight decreases seen at 18 dpi. Significant differences were measured in groups one and two compared with group three (p < 0.001) at every stage, except at 1 dpi where significant differences only were seen between groups two and three (p < 0.05). Expression of TNF-α in HLN increased significantly only at 18 dpi in groups one (p < 0.001) and two (p < 0.05) compared with group three. No significant differences were found between groups one and two at any point of infection.

In general, our results agree with the gradual increase in expression of TNF-α and IL-1β reported in a similar experiment on liver tissue, although IL-1β gene expression was highest in liver at 18 dpi, in HLN it was highest at 9 dpi^33^. This differences in expression patterns of IL-1β between liver and HLN tissues could be due to additional inflammatory reactions of the liver against excretory-secretory (ES) products of the parasite^33^ or to the different stage of the disease (18 dpi) in HLN and 16 wpi in the liver.

A preliminary study was carried out as part of experiment B to evaluate the expression of TNF-α and IL-1β in the liver. In our previous study^33^, expression of regulatory and proinflammatory cytokines was evaluated in the liver of sheep in early stages of infection with *F. hepatica*. To evaluate reference genes in liver tissue, the present study analysed tissue samples from experiment B, to compare chronically infected animals with uninfected animals.

**Figure 7:**
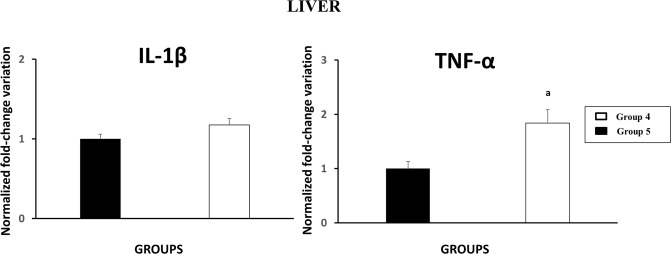
Gene expression of proinflammatory cytokines in liver. ^a^Indicates significant differences (p < 0.001) in comparison with group 5 (uninfected control).

The results of proinflammatory cytokine measurements in experiment B (16 weeks post infection) are illustrated in Fig. 7. Group four showed slightly higher IL-1β gene expression than group five (uninfected control) but the differences were not statistically significant. Expression of TNF-α was double in group four compared with group five (uninfected control), finding significant differences (p < 0.01) between them.

Fasciola hepatica is able to modulate the host immune response by stimulating suppressive cytokines such as IL-10 and TGF-β, and increasing the expression of FoxP3 which plays an important role in parasite survival^33^. This parasite is called the “master of immune modulation” due to its ability to persist in the host for years through interactions with inflammatory and immune mechanisms associated with other infections or vaccines^37^. This immune modulation results in downregulation of the Th1 immune response which normally plays an important role in host protection^38,39^, and upregulation of the Th2 immune response^10^. Although there are effector mechanisms regulated by Th2 against *Strongylus* spp. infection in sheep^40^, *F. hepatica* has developed several escape mechanisms in order to avoid the effects of Th2 immune response^41^. Polarisation of the immune response is accompanied by a suppression of the inflammatory response, where proinflammatory cytokines (TNF-α and IL1-β) are involved^42^. Some vaccine trials carried out with recombinant cathepsin L1 protease combined with Montanide adjuvant conferred protection against *F. hepatica* infection in cattle^43^, and another study proved that this protection is mediated by a Th1-like response^44^.

In a previous study following the same protocol as experiment A of the present work, expression levels of TGF-β, IL-10, FoxP3, IL-1β and TNF-α were evaluated in the liver^33^, as this is the target organ of *F. hepatica* in sheep. Increased RNA levels of FoxP3, IL-10 and TGF-β and a positive correlation between FoxP3 and IL-10 were observed, indicating that the parasite modulates the host response to facilitate its survival during early stages of disease.

To sum up, using a comprehensive statistical approach (RefFinder) that encompasses four different methods of analysis (geNorm, BestKeeper, comparative Ct method and NormFinder), we have validated a set of 10 candidate reference genes for gene normalisation using qRT-PCR in HLN and liver tissues of sheep. Stability analysis by a pairwise variation (Vn/Vn + 1) method revealed that PGK1, HSP90AA1 and GYPC were the most stable reference genes and suitable for qRT-PCR normalisation in both tissues. These three genes were validated against FoxP3, IL-10, TGF-β, TNF-α and IL-1β genes in HLN tissue, and against TNF-α and IL-1β in liver tissue. Our study is the first to evaluate and validate reference genes in sheep liver and HLN. Our findings will allow further analysis of *Ovis aries* gene expression to elucidate the role of different regulatory cytokines in the protection afforded by different antigenic cocktails. This will contribute to development of an efficient vaccine against *Fasciola hepatica*.

## Materials and Methods

### Experimental design

**Figure 8:**
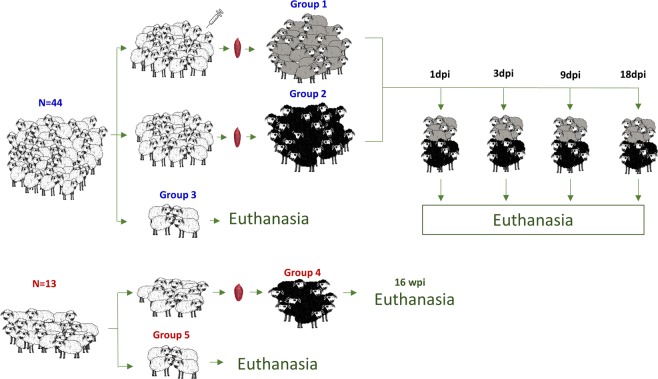
Timing of immmunisations, infections and sacrifice of groups one to five.

One-month-old female Merino sheep were obtained from a liver fluke-free farm and maintained in the experimental farm of the University of Córdoba for six months. Animals were tested monthly^10^ for parasite eggs by fecal sedimentation to confirm absence of *F. hepatica* infection. We carried out two different experiments showed in Fig. 8, both were approved by the Bioethics Committee of the University of Cordoba (approval number 1118) and conducted in accordance with European (2010/63/UE) and Spanish (RD 1201/2005) directives on animal experimentation.

In the first experiment (A), 44 sheep were divided into three principal groups: group one (n = 20) was composed of animals immunised subcutaneously with two doses 4 weeks apart of a vaccine (CL1) comprise of 100 µg recombinant CL1 from *F. hepatica* (kindly provided by Professor Dalton, Queen’s University Belfast, UK) in 2 ml of Montanide ISA 70 VG (Seppic, Puteaux, France). Sheep were additionally infected with 200 metacercariae (mc) of the South Gloucester strain of *F. hepatica* (Ridgeway Research Ltd, UK) administered in a single dose. Sheep in group two (n = 20) were infected with the same dose of mc only, and animals in group three (n = 4) were used as uninfected controls (UC). Five animals from groups one and two were sacrificed at 1, 3, 9 and 18 dpi. Previous reports described no differences in fluke burden between animals immunised with recombinant cathepsin L1 and subsequently infected, animals immunised with adjuvant Montanide ISA 70VG only and unimmunised infected sheep^10^.

In the second experiment (B), 13 sheep were divided into two groups: group four (n = 9) was infected with a single dose of 150 mc and group five (n = 4) was used as the UC. Infected animals were sacrificed at 16 weeks post-infection.

### Pathology

Sheep were subjected to necropsy where the diaphragmatic and visceral surfaces of the livers were photographed. Tissue samples were then collected from the surface of the left lobe, fixed in 10% neutral-buffered formalin for 24 hours and embedded in paraffin wax. For each animal, between four and nine tissue samples were collected from affected areas when present. Four micron thick tissue sections were stained with haematoxylin and eosin (for histopathology).

### Total RNA isolation and cDNA synthesis

Tissue samples from HLN of animals in experiment A, and three slice from the left hepatic lobe of sheep in experiment B, were collected for qRT-PCR. Samples were rinsed in diethyl pyrocarbonate (DEPC; Applichem Panreac, Gatersleben, Germany) treated water and flash frozen in liquid nitrogen. Before use, samples were individually snap freeze and stored at −80 °C.

Total RNA was isolated from 300 mg of ground tissue that had been homogenised in 1.5 ml of TRIzol reagent (Ambion, Life Technologies, CA, USA) as previously described^10^. The quality of RNA was determined by spectrophotometry, and its integrity was evaluated using an Agilent 2100 Bioanalyser (Agilent Technologies, CA, USA). The iScript cDNA Synthesis kit (BioRad, CA, USA) was used to generate cDNA according to the manufacturer’s instructions with a first step of priming at 25 °C for 10 min, a second step of retrotranscription at 46 °C for 20 min and a last step for retrotranscriptase inactivation at 95 °C for 1 min.

### Reference gene selection, primer design and qRT-PCR conditions

Ten putative reference genes were selected to be analysed in this work. Primer pairs used for specific amplification and quantification of these genes are listed in Table 1.

Primers were designed using sheep gene sequences deposited in the GenBank database (NCBI, https://www.ncbi.nlm.nih.gov/genbank/) with OLIGO 7 Primer Analysis Software (Molecular Biology Insights, Inc., http://www.oligo.net) as previously described^10,45^. In addition to being free of hairpins and duplex structures, primers were required to have a high Tm (≥70 °C) to ensure specificity.

Transcript quantification was carried out using 50 ng of cDNA per reaction and the SsoAdvanced Universal SYBR Green Supermix (BioRad) kit, according to the manufacturer’s instructions and using a two-step 94/68 °C protocol for PCR reactions. All samples were quantified in triplicate in a MyiQ2 Two-Colour Real-Time PCR Detection System (BioRad) as previously described^10^. A melting curve analysis from 65 °C to 95 °C was applied to all PCR reactions to ensure specificity of amplification.

A 10-fold dilution series (resulting in a concentration range from 20 to 2 × 10^5^ pg) of cDNA that was generated from an RNA pool made by mixing equal amounts of total RNA from any animal and tissue was created. The cDNA was used to generate a standard curve and determine the qRT-PCR efficiency (E) for each gene using a linear regression model^20^. The corresponding E values were calculated from the slope(s) of the standard curve according to equation: E = 10^(−1/slope)^−1.

Baseline correction was performed using the automatic function of MyiQ2 Software. Transcript abundance was defined as the number of amplification cycles required for each gene to reach a fixed threshold in the exponential phase of PCR reaction^46^. An inter-run calibrator (IRC) RNA sample with a known quantity of A170 gene transcripts was included in each plate to set the threshold and determine Ct values, using the Single Threshold mode of MyiQ2.

### Expression stability analysis of candidate reference genes

The stability of each candidate reference gene was evaluated using the algorithms geNorm^22^, NormFinder^21^, BestKeeper^20^ and the comparative ∆Ct method^23^ integrated into the comprehensive web-based analysis tool RefFinder^19^. GeNorm determines the most stable reference genes and the optimal number of genes needed for accurate normalisation. This algorithm transformed qRT-PCR Ct values obtained from MyiQ2 into linear relative values using the comparative ∆Ct method.

The Ct values obtained from qRT-PCR using MyiQ2 were transferred to Microsoft Excel for calculation of linear relative values (keeping the lowest relative quantity for each gene as one). Values were then imported into geNorm to calculate gene expression stability value (M) and the pairwise variation value (V) for each reference gene, and to rank genes according to their M value. The cut-off M value was set at 1.5, with a lower M value indicating more stable expression. The MyiQ2 software was used to determine the optimal number of reference genes for normalisation by analysing the pairwise variation (Vn/Vn + 1). A cut-off ≤0.15 indicated that inclusion of additional reference genes was not necessary. The linear relative quantities calculated using geNorm were also used as input data for NormFinder to calculate expression stability and rank candidate reference genes according to intra- and intervariation. NormFinder also determined the standard deviation (SD) and selected the genes with the lowest M values (lowest intra- and inter-group variation). BestKeeper directly utilised Ct values obtained from the thermocycler software to evaluate the SD, p values and correlation coefficient of each gene. Genes with lower SD values were considered better potential reference genes. RefFinder is a comprehensive tool which also directly uses the Ct values obtained from the thermocycler software to generate an overall ranking of gene stability, through calculation of the weighted geometric mean of every tested gene calculated by each individual algorithm, including the comparative ∆Ct method.

### Validation of Identified Reference Genes

Five genes, FoxP3, IL-10, TGF-β, TNF-α and IL-1β were selected as target genes to validate the reliability of identified reference genes in HLN and/or liver tissues. Average Ct values were calculated from four to nine biological replicates in each experimental condition, and from three technical replicates used for relative expression analyses. Gene expression profiles of these five target genes were normalised using the most stable candidate reference gene(s). Relative quantification of target genes in different samples was carried out using the comparative ∆∆Ct method as described in the following equation: Fold variation = (1 + E)^−ΔΔCt^.

### Statistical analyses

Comparison of data obtained from experimental groups with control group data was carried out with Dunnett’s test using InStat v.3.05 (GraphPad software Inc., CA, USA). Kolmogorov-Smirnov and Bartlett normality tests were then performed, and where samples passed these normality tests, a Bonferroni multiple comparison test was used to compare two treatments with each other. When samples did not pass the normality tests the Kruskal-Wallis nonparametric test was used. P values of < 0.05 were considered to be statistically significant.

## Supplementary information

Dataset 1

## Data Availability

The datasets generated during and/or analysed during the current study are available from the corresponding author on reasonable request.
