# Supplementary material for: Identification of reference genes for real-time PCR cytokine gene expression studies in sheep experimentally infected with Fasciola hepatica
Source: Sci Rep. 2019 Feb 6;9:1485. doi: 10.1038/s41598-018-37672-7 (PMC6365638; doi:10.1038/s41598-018-37672-7)
Supplement: Supplementary file 1 — Dataset 1 [file 41598_2018_37672_MOESM1_ESM.pdf]

Identification of reference genes for real-time PCR cytokine gene expression studies in sheep experimentally infected with *Fasciola hepatica*

I.L. Pacheco[a], N. Abril[b], R. Zafra[c], N. Morales-Prieto[b], V. Molina Hernández[a], M.T. Ruiz[a], R. Perez-Caballero[c], A. Martínez-Moreno[c], J. Pérez[a*]

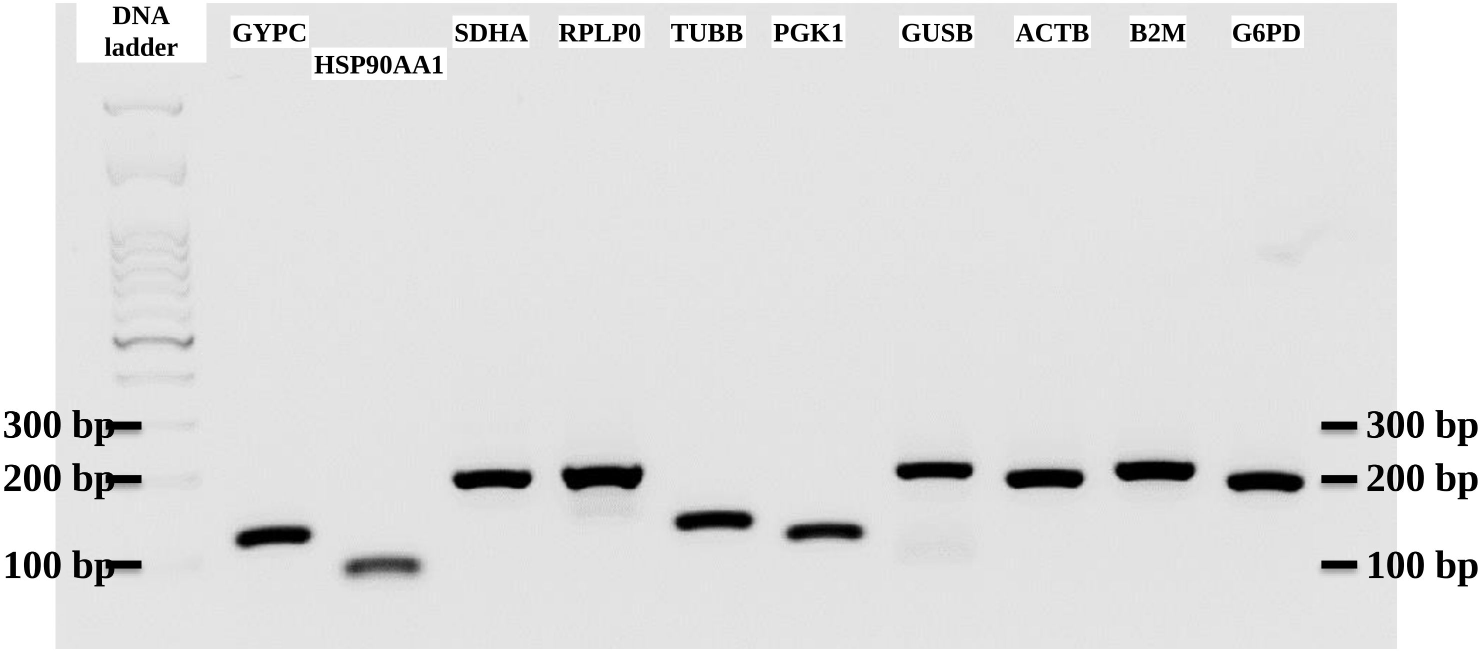

Supplementary Figure 1. Agarose gel (3%) electrophoresis showing amplicon size for ten candidate reference genes.

HLN										LIVER									
Hsp90	GUSB	GYPC	Tubβ	B2M	SDHA	RPLPO	G6PD	Pgk1	ACTB	Hsp90	GUSB	GYPC	Tubβ	B2M	SDHA	RPLPO	G6PD	Pgk1	ACTB
17,68	23,82	21,91	24,50	12,88	20,42	14,32	21,17	19,60	15,00	19,70	23,12	23,69	32,94	15,40	20,93	18,61	26,79	21,70	18,97
17,37	23,68	21,71	24,56	12,70	20,97	13,45	21,32	19,65	14,76	19,41	22,61	23,37	31,75	15,24	20,67	18,25	25,95	20,47	18,86
17,33	23,64	21,38	24,72	12,92	20,94	13,56	21,58	19,85	14,73	19,35	25,64	23,59	32,62	15,20	20,76	18,46	26,24	21,52	18,92
17,03	23,78	20,69	24,00	13,13	21,36	14,51	22,16	20,06	14,52	18,46	22,63	23,72	31,13	14,94	20,52	18,67	26,36	21,40	18,75
16,94	23,79	21,02	24,20	13,14	21,45	14,61	21,86	20,27	14,55	17,72	22,93	23,63	31,42	15,01	20,86	18,83	26,25	21,86	18,99
17,09	23,47	20,94	24,37	13,09	21,23	14,31	21,81	20,31	14,59	18,01	22,84	23,65	31,75	14,93	20,67	18,78	26,09	21,85	18,58
17,01	23,70	20,46	24,30	13,49	20,93	14,04	21,73	20,09	14,35	18,44	22,82	23,30	31,14	15,59	20,12	16,93	25,98	21,05	16,86
17,33	23,67	20,45	24,23	13,20	20,90	14,03	21,88	19,72	14,13	18,23	22,50	22,98	31,14	15,37	20,41	17,03	25,68	21,03	16,76
17,28	23,71	20,87	24,39	13,12	20,86	14,08	21,94	20,24	14,22	18,19	22,54	23,03	30,88	15,34	20,35	16,97	25,88	20,95	16,75
17,01	23,38	20,83	23,88	13,16	20,86	13,79	21,42	19,79	13,88	18,31	22,88	22,82	31,70	15,62	20,76	18,13	25,76	21,66	17,70
16,95	22,91	20,75	24,01	13,17	21,02	14,03	21,64	19,86	13,94	18,52	22,76	22,61	31,00	15,00	20,77	18,31	25,88	20,92	17,72
17,12	23,32	20,92	24,46	13,21	21,03	13,90	21,55	19,91	14,01	18,84	22,34	23,06	32,02	15,70	20,67	18,10	26,31	21,61	17,84
17,04	23,58	21,47	24,27	13,26	20,99	14,09	21,96	20,02	14,96	17,73	22,35	22,70	30,33	15,08	20,07	16,51	24,22	20,45	16,36
16,91	23,01	21,31	24,59	13,10	20,91	14,08	22,01	19,94	14,85	17,38	21,96	22,54	32,09	15,09	19,67	16,41	24,19	20,42	16,36
17,60	23,59	21,40	24,31	12,95	21,07	14,34	21,76	20,01	15,06	17,80	22,32	22,62	31,53	15,13	19,89	16,56	24,20	20,63	16,40
16,82	22,92	21,00	23,79	13,21	20,34	14,29	21,98	20,11	14,95	18,04	20,94	22,78	31,52	15,35	19,65	16,82	25,61	20,94	16,72
16,34	23,12	21,04	23,46	12,79	20,77	14,28	21,80	19,98	14,86	17,71	20,87	22,71	31,24	14,87	19,42	16,30	24,97	20,75	16,77
16,70	23,20	20,93	23,77	12,91	21,09	14,16	21,72	20,16	14,88	18,09	21,44	23,12	30,83	15,00	19,47	16,76	25,08	20,67	17,14
16,57	22,76	20,40	22,92	12,41	20,92	14,10	21,82	19,49	17,74	18,14	22,74	22,81	29,95	14,80	19,68	16,73	24,85	20,86	16,63
16,39	22,95	20,56	22,68	13,64	21,37	14,14	21,97	19,86	14,27	17,86	21,80	22,90	30,52	14,80	19,61	16,79	25,05	21,29	16,66
16,48	22,85	20,30	22,76	13,29	21,18	14,09	22,01	19,71	14,48	17,89	21,66	22,73	29,86	14,98	19,67	16,89	25,07	20,98	16,62
17,07	23,22	20,75	23,54	16,37	20,12	14,07	22,13	19,85	14,31	18,16	22,16	22,81	30,97	14,59	19,38	17,21	25,25	20,68	16,36
16,62	23,48	20,35	23,61	13,52	20,50	13,90	22,07	19,80	14,17	18,02	22,02	22,94	31,10	14,58	19,49	16,99	25,46	20,36	16,36
16,84	23,39	20,64	23,30	13,68	20,66	13,92	22,01	19,79	14,17	18,00	21,97	22,94	30,83	14,73	19,44	16,89	25,60	20,57	16,40
17,26	23,74	21,19	24,77	13,28	21,54	13,98	22,21	20,28	14,65	17,93	22,36	22,97	31,06	15,31	19,86	17,03	25,05	21,32	16,72
17,24	24,07	21,19	24,97	13,11	21,52	13,98	22,07	20,38	14,73	17,87	22,45	23,15	31,02	15,40	20,10	17,25	25,21	21,35	16,77
17,38	24,24	21,22	24,91	13,05	21,40	14,13	23,85	20,55	14,87	17,99	23,30	23,22	31,32	15,58	19,84	17,20	25,34	21,60	17,14
17,28	23,88	21,37	25,01	13,44	21,49	14,11	22,00	20,25	14,84	18,18	22,28	22,84	30,29	14,72	19,67	17,74	25,33	20,94	16,63
17,32	24,21	21,30	24,91	13,43	21,49	14,18	21,79	20,19	14,89	18,25	22,49	23,05	31,11	14,77	20,13	17,59	25,14	21,05	16,66
16,29	23,69	21,09	24,57	12,67	21,33	14,20	21,76	20,08	14,88	18,07	22,41	22,79	30,87	15,04	19,27	17,67	25,31	20,94	16,62
17,13	22,76	21,02	25,62	12,92	21,23	13,87	21,71	20,13	14,56	18,03	22,61	22,99	31,95	15,03	18,95	16,16	23,16	20,30	16,36
17,11	22,85	20,79	25,24	12,53	21,19	13,91	21,76	20,00	14,61	18,26	22,68	22,93	31,85	14,85	19,04	15,98	23,70	19,86	16,36
17,01	22,77	20,94	25,71	13,34	21,24	14,08	21,49	20,10	14,67	18,47	22,71	23,30	32,71	15,00	18,98	16,10	23,56	20,09	16,40
17,45	23,01	20,92	23,43	13,21	19,69	14,04	21,63	20,00	14,87	18,57	22,36	22,70	31,73	15,23	18,81	15,71	24,71	20,97	16,72
17,84	22,90	20,79	23,40	12,78	19,83	13,88	21,85	19,96	14,73	17,93	21,85	22,93	31,42	15,08	18,66	15,39	24,57	20,77	16,77
17,95	23,08	20,59	23,55	12,85	19,92	13,97	21,74	19,87	14,77	18,16	21,91	23,10	31,19	15,16	18,81	15,47	24,83	20,83	17,14
16,75	23,49	20,78	24,37	12,67	20,12	14,14	21,62	19,80	14,71	17,93	21,91	23,11	31,61	15,00	19,58	15,92	25,19	20,23	16,63
16,76	23,79	20,78	24,61	13,21	20,16	14,15	21,59	19,79	14,75	18,13	22,34	23,36	31,51	15,07	19,51	16,18	25,13	20,34	16,66
16,26	23,80	20,83	24,74	12,83	21,09	14,16	22,01	19,73	14,71	18,07	22,60	23,51	31,32	15,38	19,48	16,44	25,30	20,09	16,62
17,32	23,25	20,28	23,58	12,88	19,07	13,76	21,07	19,14	14,14	17,98	22,96	22,43	30,86	14,42	20,43	17,18	24,71	21,48	16,36
16,25	23,13	20,52	23,82	12,76	19,50	13,79	21,18	19,30	14,07	18,08	22,56	22,47	30,73	14,21	20,44	17,32	24,82	22,14	16,36
16,16	22,87	20,35	23,75	13,32	19,82	13,67	21,07	18,96	13,91	18,02	22,27	22,20	30,52	14,50	20,21	17,03	24,34	21,37	16,40
16,90	22,96	20,45	24,19	13,91	20,95	13,70	22,15	19,78	14,40	17,90	22,12	22,96	31,91	15,38	19,36	16,63	25,05	20,71	16,72
17,00	22,58	20,06	24,08	13,19	20,96	13,56	22,06	19,67	14,43	17,89	22,11	22,98	31,93	14,62	19,21	16,34	24,80	20,57	16,77
16,70	22,59	20,43	24,50	13,15	20,84	13,63	21,95	19,74	14,37	17,92	21,73	23,03	31,87	15,21	19,43	16,58	25,07	20,52	17,14
17,00	23,55	20,66	25,35	12,88	20,89	14,07	21,65	20,04	14,73	17,83	22,69	22,93	31,84	15,31	19,60	15,73	24,15	20,78	16,63
17,35	23,66	20,76	25,60	12,61	21,20	14,08	21,23	20,11	14,74	17,87	22,37	22,91	30,93	15,53	19,70	16,02	24,22	20,90	16,66
17,44	23,77	21,11	25,19	12,38	21,43	14,24	21,48	20,46	14,87	17,56	22,58	23,13	32,07	15,00	19,93	16,16	24,65	21,16	16,62
16,65	23,10	20,26	23,38	12,61	18,69	13,67	21,00	19,45	14,04	17,98	21,78	22,91	30,68	14,84	19,32	16,02	24,47	19,99	15,80
16,55	23,06	20,23	23,49	12,69	18,81	13,80	20,65	19,20	13,95	17,95	21,80	23,08	29,71	14,61	19,02	15,87	24,15	19,83	15,91
15,81	23,01	20,36	23,22	12,84	18,68	13,72	20,89	19,26	14,13	17,90	22,07	22,69	29,27	14,58	18,79	15,94	24,54	20,39	15,82
15,81	23,00	21,04	24,66	12,88	19,75	14,65	20,85	19,54	14,58	17,99	22,29	22,99	29,49	15,15	19,21	16,20	23,83	20,35	15,78
15,93	23,84	20,92	24,71	12,68	20,91	14,66	21,00	19,49	14,35	17,79	22,29	23,54	30,76	15,00	19,61	15,95	24,11	20,66	15,64
16,52	23,89	21,23	24,47	12,49	21,01	14,65	20,71	19,98	15,06	17,98	22,76	22,81	31,89	15,39	19,36	16,48	24,17	20,84	15,92
16,01	23,58	20,00	24,36	13,30	20,70	13,95	21,23	19,64	13,97	17,66	21,89	22,48	29,31	15,01	19,10	15,67	23,81	20,59	15,35
16,38	23,30	20,04	23,88	12,80	20,66	13,95	21,06	19,54	14,01	17,56	21,64	22,34	29,90	15,11	19,35	15,80	23,78	20,58	15,42
16,26	23,43	19,82	23,94	12,81	20,69	14,10	21,45	19,73	13,96	17,95	21,93	22,65	29,51	15,06	19,74	15,69	23,79	20,54	15,68
16,29	23,18	20,03	23,07	13,00	20,00	13,98	21,26	18,87	13,82	17,74	21,85	22,79	29,51	13,32	18,69	16,09	23,80	20,17	15,47
15,44	23,34	19,97	23,53	12,84	19,94	14,01	21,47	18,82	13,84	17,94	22,22	22,47	30,00	13,92	19,39	16,49	23,73	20,00	15,67
15,55	23,30	20,19	23,17	13,21	20,12	14,20	21,52	18,87	13,95	17,95	22,44	22,60	29,82	14,16	19,52	16,48	24,11	20,40	15,51
16,29	23,48	20,68	24,60	13,04	20,32	14,24	20,90	19,41	13,91	17,90	25,03	22,17	30,74	15,21	19,94	16,63	23,91	21,21	15,15
16,60	23,67	20,93	25,33	12,68	20,69	14,39	20,92	19,49	14,16	18,16	21,82	22,44	30,96	15,80	20,64	16,76	24,05	21,25	15,55
16,21	23,53	20,62	25,00	12,29	20,55	14,30	20,96	19,42	13,96	17,94	21,73	21,91	31,18	15,48	20,11	16,48	23,86	21,33	15,19
16,37	23,40	20,37	23,18	12,37	18,77	14,00	21,50	18,91	14,14	17,89	22,18	22,79	28,53	15,00	19,78	16,49	23,96	20,80	15,91
15,60	23,60	20,10	23,19	12,68	18,53	13,99	21,49	19,31	14,05	17,57	22,01	22,89	29,66	15,14	19,76	16,53	23,85	20,84	15,87
15,91	23,38	20,39	23,13	12,75	18,65	13,95	20,77	19,46	14,04	17,79	21,52	23,14	29,98	14,91	19,78	16,47	24,03	20,87	15,72
16,45	23,14	20,67	24,37	12,87	20,36	13,79	20,46	19,34	14,00	16,88	21,17	21,65	30,41	15,14	18,98	15,17	22,75	20,24	15,16
16,68	23,15	20,36	24,52	13,00	20,40	13,95	20,36	19,59	14,01	17,17	20,83	21,90	30,68	14,55	19,81	14,96	22,74	20,15	15,19
16,45	23,14	20,42	24,89	12,86	20,34	14,10	20,47	19,60	14,10	17,25	20,86	21,93	30,32	15,15	19,38	15,16	22,70	20,27	15,29
15,58	22,82	20,62	22,77	12,88	18,25	14,09	21,11	17,65	13,67	17,59	22,29	21,94	26,79	12,97	20,18	15,03	19,60	20,17	14,68
15,75	22,85	20,29	22,73	12,37	18,38	13,88	20,90	17,13	13,64	17,70	22,45	22,08	26,73	12,80	19,89	14,60	19,40	19,67	14,33
15,61	22,79	20,40	22,76	12,22	19,01	13,87	21,33	16,92	13,56	17,59	22,07	21,81	27,64	12,87	20,00	14,59	19,08	19,08	14,26
15,29	23,05	20,24	22,81	12,64	18,46	13,83	26,97	16,21	13,50	17,49	20,87	22,66	30,79	14,31	19,29	15,43	20,98	20,14	15,34
15,72	22,88	20,16	23,72	12,53	18,85	13,72	37,54	16,18	13,67	17,64	20,91	22,77	30,32	14,18	18,81	15,31	21,26	20,18	15,91
15,77	23,17	20,45	23,35	12,77	19,59	14,04	22,11	16,49	13,82	17,85	21,03	22,98	29,89	14,10	19,09	15,77	21,67	20,58	16,05
16,32	23,86	20,92	23,14	12,80	18,73	14,24	21,00	17,78	13,79	17,54	22,25	22,67	27,48	14,75	19,29	15,48	22,29	20,39	15,35
15,74	23,71	20,89	23,35	12,96	19,08	14,05	21,36	18,05	13,80	17,70	21,82	22,89	28,36	15,19	19,39	15,75	22,09	20,47	15,17
16,06	24,00	20,83	23,11	12,76	19,78	14,09	21,23	17,80	13,90	17,76	22,24	22,82	27,30	14,54	19,34	15,49	21,72	20,01	15,48
16,72	23,41	20,49	24,39	12,94	20,56	14,39	21,05	18,77	14,10	17,68	22,74	22,83	25,67	13,41	19,20	15,12	19,79	20,11	14,81
16,76	23,52	20,59	24,22	12,93	20,87	14,23	21,30	18,12	14,03	17,53	22,58	22,75	25,79	13,72	18,93	14,85	19,74	19,97	14,83
16,35	23,74	20,65	24,85	12,93	20,79	14,34	21,26	17,71	14,07	17,74	23,34	22,99	26,00	13,66	19,21	15,15	19,93	20,83	14,90
16,17	23,73	20,82	24,09	12,88	20,68	13,93	21,03	17,47	14,02	17,86	22,78	22,30	26,14	14,66	19,07	15,41	20,75	21,47	15,01
15,84	23,66	20,74	23,81	12,87	20,45	14,04	21,04	18,45	14,00	18,06	22,22	22,16	26,80	14,68	19,72	15,51	21,35	20,82	14,44
15,76	23,60	20,49	24,05	12,88	20,21	13,98	20,87	18,98	13,99	17,83	22,55	21,89	26,78	15,01	19,87	15,31	21,68	21,07	14,43

Supplementary Table S1. Ct values obtained for each candidate gene in both HLN and liver tissues.

HLN+liver

Statistical parameter	*PGK1*	*B2M*	*G6PD*	*SDHA*	*RPLP0*	*GUSB*	*ACTB*	*HSP90*	*GYPC*	*TUBB*
Observations	168	168	168	168	168	168	168	168	168	168
Minnimum value	17,470	12,290	19,080	18,250	13,450	20,830	13,500	15,290	19,820	22,680
Maximun value	22,140	16,370	26,790	21,540	18,830	25,640	18,990	19,700	23,720	32,940
1st Quartil	19,790	12,938	21,465	19,390	14,080	22,290	14,208	16,673	20,688	24,165
Median	20,235	14,170	22,110	20,035	15,240	22,850	14,885	17,470	21,760	25,690
3rd Quartil	20,840	15,045	24,823	20,763	16,565	23,415	16,360	17,933	22,833	30,838
Average	20,273	14,041	23,012	20,052	15,463	22,813	15,317	17,293	21,753	27,205
Variance (n-1)	0,661	1,181	3,791	0,680	2,289	0,661	1,647	0,737	1,302	11,617
Standard deviation (n-1)	0,813	1,087	1,947	0,825	1,513	0,813	1,283	0,858	1,141	3,408

HLN

Statistical parameter	*PGK1*	*B2M*	*G6PD*	*SDHA*	*RPLP0*	*GUSB*	*ACTB*	*HSP90*	*GYPC*	*TUBB*
Observations	84	84	84	84	84	84	84	84	84	84
Minnimum value	17,470	12,290	20,360	18,250	13,450	22,580	13,500	15,290	19,820	25,670
Maximun value	21,860	16,370	26,790	21,540	18,830	24,240	17,740	17,950	21,910	32,940
1st Quartil	19,483	12,788	21,178	20,090	13,950	23,040	13,998	16,240	20,398	29,850
Median	19,800	12,955	21,640	20,690	14,080	23,395	14,195	16,665	20,685	30,845
3rd Quartil	20,110	13,265	22,010	21,013	14,293	23,683	14,730	17,075	20,933	31,443
Average	19,817	13,243	22,005	20,431	14,486	23,368	14,356	16,618	20,699	30,341
Variance (n-1)	0,596	0,706	2,345	0,754	1,673	0,153	0,311	0,379	0,172	2,885
Standard deviation (n-1)	0,772	0,840	1,531	0,868	1,293	0,391	0,558	0,616	0,414	1,699

Liver

Statistical parameter	*PGK1*	*B2M*	*G6PD*	*SDHA*	*RPLP0*	*GUSB*	*ACTB*	*HSP90*	*GYPC*	*TUBB*
Observations	84	84	84	84	84	84	84	84	84	84
Minnimum value	19,080	12,800	19,080	18,660	14,590	20,830	14,260	16,880	21,650	22,680
Maximun value	22,140	15,800	26,790	20,930	18,830	25,640	18,990	19,700	23,720	25,710
1st Quartil	20,348	14,618	23,723	19,290	15,725	21,880	15,503	17,740	22,618	23,453
Median	20,730	15,000	24,280	19,610	16,455	22,290	16,360	17,930	22,835	24,140
3rd Quartil	21,050	15,203	25,195	20,018	16,975	22,603	16,720	18,083	23,035	24,593
Average	20,728	14,840	24,018	19,673	16,439	22,259	16,278	17,969	22,807	24,068
Variance (n-1)	0,313	0,380	3,232	0,323	1,003	0,555	1,133	0,179	0,200	0,576
Standard deviation (n-1)	0,560	0,616	1,798	0,568	1,001	0,745	1,065	0,423	0,448	0,759

Supplementary Table S2.

Comparison of candidate reference gene expression stabilities by descriptive statistics using the XLStat v.19.4.45191 software (Addisoft) to calculate the statistical parameters of Ct values. Data in this table were used to obtain the box-plots shown in Figure 3.
